# Use of Hybrid Assistive Limb (HAL^®^) for a postoperative patient with cerebral palsy: a case report

**DOI:** 10.1186/s13104-018-3311-z

**Published:** 2018-03-27

**Authors:** Yuki Mataki, Hiroshi Kamada, Hirotaka Mutsuzaki, Yukiyo Shimizu, Ryoko Takeuchi, Masafumi Mizukami, Kenichi Yoshikawa, Kazushi Takahashi, Mayumi Matsuda, Nobuaki Iwasaki, Hiroaki Kawamoto, Yasuyoshi Wadano, Yoshiyuki Sankai, Masashi Yamazaki

**Affiliations:** 1 Department of Orthopaedic Surgery, Ibaraki Prefectural University of Health Sciences Hospital, 4773 Ami, Inashiki-gun, Ibaraki 300-0331 Japan; 2 Department of Orthopaedic Surgery, University of Tsukuba, 1-1-1 Tennoudai, Tsukuba, Ibaraki 305-8575 Japan; 3 Department of Rehabilitation Medicine, University of Tsukuba Hospital, 2-1-1 Amakubo, Tsukuba, Ibaraki 305-8576 Japan; 4 Center for Medical Sciences, Ibaraki Prefectural University of Health Sciences, 4669-2 Ami, Inashiki-gun, Ibaraki 300-0394 Japan; 5 Department of Physical Therapy, School of Health Sciences, Ibaraki Prefectural University of Health Sciences, 4669-2 Ami, Inashiki-gun, Ibaraki 300-0394 Japan; 6 Department of Physical Therapy, Ibaraki Prefectural University of Health Sciences Hospital, 4733 Ami, Inashiki-gun, Ibaraki 300-0331 Japan; 7 Information and Systems, Faculty of Engineering, University of Tsukuba, Tsukuba, Japan; 8 Cybernic Research Center, University of Tsukuba, Tsukuba, Japan

**Keywords:** Hybrid Assistive Limb (HAL), Cerebral palsy, Operation

## Abstract

**Background:**

The Hybrid Assistive Limb (HAL^®^) is an exoskeleton wearable robot suit that assists in voluntary control of knee and hip joint motion. There have been several studies on HAL intervention effects in stroke, spinal cord injury, and cerebral palsy. However, no study has investigated HAL intervention for patients with cerebral palsy after surgery.

**Case presentation:**

We report a case of using HAL in a postoperative patient with cerebral palsy. A 15-year-old boy was diagnosed with spastic diplegia cerebral palsy Gross Motor Function Classification System level IV, with knee flection contracture, equinus foot, and paralysis of the right upper extremity with adduction contracture. He underwent tendon lengthening of the bilateral hamstrings and Achilles tendons. Although the flexion contractures of the bilateral knees and equinus foot improved, muscle strength decreased after the soft tissue surgery. HAL intervention was performed twice during postoperative months 10 and 11. Walking speed, stride, and cadence were increased after HAL intervention. Post HAL intervention, extension angles of the knee in stance phase and hip in the pre-swing phase were improved. In the gait cycle, the proportion of terminal stance in the stance and swing phase was increased.

**Conclusions:**

Hybrid Assistive Limb intervention for postoperative patients with cerebral palsy whose muscle strength decreases can enhance improvement in walking ability. Further studies are needed to examine the safety and potential application of HAL in this setting.

**Electronic supplementary material:**

The online version of this article (10.1186/s13104-018-3311-z) contains supplementary material, which is available to authorized users.

## Background

**Fig. 1:**
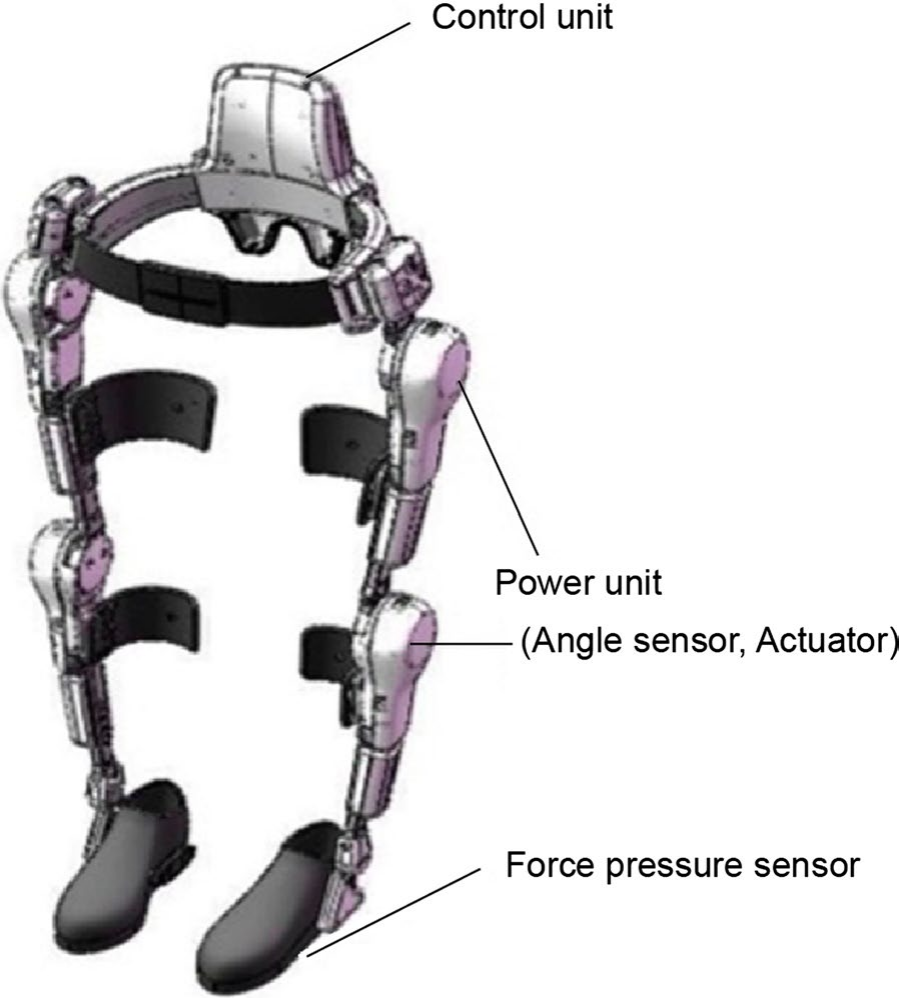
Hybrid Assistive Limb (HAL^®^)

The Hybrid Assistive Limb (HAL^®^) is a wearable robot suit that assists in voluntary control of knee and hip joint motion [1]. The HAL detects the bioelectric signals generated by patients’ muscle activities and/or force-pressure signals caused by patients’ weight shifts. The bioelectric signals are detected from the hip extensor muscle, hip flexion muscle, knee extensor muscle, and knee flexion muscle by applying electrodes. Electrodes are often applied to the gluteus, rectus femoris, quadriceps femoris, and hamstrings. Power units on the hip and knee joints on both sides consist of angular sensors and actuators, and the control system consists of cybernic voluntary control (CVC) and cybernic autonomous control (CAC) subsystems (Fig. 1). HAL^®^ differs from other robots in that it provides motion according to the wearer’s voluntary drive. Other robots use autonomously generated predefined motion for users.

Hybrid Assistive Limb intervention improved walking ability and balance in chronic stroke patients [2–4]. Kawamoto et al. [2] reported that walking speed, stride, and cadence in the 10 m-walk test and Berg balance scale increased in chronic stroke patients. In the acute stroke phase, HAL intervention may improve outcomes in selected patients [5–10]. Moreover, there have been several studies of HAL intervention effects in chronic spinal cord injury (SCI) [11–13]. Recently, some authors have reported the use of HAL in the acute phase or early postoperative period following SCI [14–16]. HAL intervention for acute and postoperative SCI patients enhances improvement in walking ability. The arrangement of the interactive feedback through HAL is postulated to efficiently excite the remaining corticospinal tract in the injured brain and spinal nerves and enhance the effects of physical therapy. Additionally, the cycle of voluntary movement from afferent signals in the brain is postulated to heighten the excitability of the existing intracortical network and promote the formation of new networks.

Cerebral palsy is associated with abnormal generation of bioelectrical signals in the lower limbs associated with brain damage. Patients with spastic cerebral palsy who could not walk alone gained gait ability by wearing HAL; therefore, Taketomi et al. [17, 18] indicated that HAL is an effective method for walking and stair ascent assistance for cerebral palsy patients. However, no report of HAL intervention has been described in a patient with cerebral palsy after surgery. We report a case of HAL intervention in a patient with cerebral palsy after tendon lengthening surgery of the bilateral hamstrings and Achilles tendons. In this study, although the flexion contracture of the bilateral knees and equinus foot improved, the muscle strength of bilateral knee flexion and ankle dorsiflexion decreased after tendon lengthening of the bilateral hamstrings and Achilles tendons. Improvement of joint range after surgery did not immediately lead to improvement in walking ability. HAL intervention with a CVC system can potentially improve walking ability due to enhanced range of movement, muscle strength, and feedback within the neurological system throughout the gait cycle. Patients with cerebral palsy and abnormal gait may be able to learn a more normal walking pattern with HAL intervention.

## Case presentation

A 15-year-old boy was diagnosed with spastic diplegia cerebral palsy, Gross Motor Function Classification System level IV, knee flexion contracture, equinus foot, and paralysis of right upper extremity with adduction contracture. His height was 160.0 cm and his weight was 50.2 kg. He underwent tendon fractional release of the bilateral tendons of the semitendinosus muscle, semimembranosus muscle, and biceps femoris muscle. Achilles tendon lengthening was performed on the right equinus foot using Vulpius elongation. His lower limbs were fixed with above knee plaster splints in the knee extended position and intermediate ankle position 2 weeks after surgery, and he subsequently used short leg braces. Preoperative passive range of joint motion values were as follows: right knee 35–130/left knee 20–130, ankle dorsiflexion with knee extension (DKE) 15/15, and ankle dorsiflexion with knee flexion (DKF) 35/35. At 6 postoperative months, range of joint motion was right knee 15–130/left knee 10–130, DKE 15/10, and DKF 25/20. Knee extension range and DKF improved postoperatively. Preoperative manual muscle testing showed the following: knee extension 3/3, knee flexion 4/4, ankle dorsiflexion 1/1, and ankle plantar flexion 1/1. Before surgery, he had begun crawling in the house. Before HAL intervention, he could not move with crawling because of postoperative muscle weakness. It was thought that muscle weakness was due to disuse muscle atrophy after surgery.

Hybrid Assistive Limb intervention was administered twice during postoperative months 10 and 11 in an outpatient department. Normal outpatient physical therapy was carried out in combination. The patient used the HAL for clinical study type S size (target 145–165 cm). A walking device (All-in-One Walking Trainer; Healthcare Lifting Specialist, Denmark) with a harness was used for safety, and the HAL intervention consisted of walking with the assistance of two physical therapists (Additional files 1 and 2). The HAL intervention session lasted 60 min, including rests (10 min) and time for attachment/detachment (20 min). The HAL suit has a hybrid control system comprising the CVC and CAC. The CVC mode of the HAL suit can support the patient’s voluntary motion by providing assistive torque to each joint according to voluntary muscle activity. This study used the CVC mode, which allows the operator to adjust the degree of physical support to the patient’s comfort and gradually reduce support as training progresses. Functional ambulation was assessed with the 10 m-walk test without wearing HAL and video analysis preoperatively and pre- and post- each HAL intervention (Additional files 3 and 4). We analyzed one gait cycle of the patient with Dartfish. Walking speed, stride, and cadence in the 10 m-walk test were analyzed. We took a walking video from the sagittal plane and analyzed the video in slow motion, pausing the image to measure the joint angle using the Dartfish Team Pro ver5.5. The angle of the hip, knee, ankle, and trunk in walking and in the gait cycle was analyzed using video. Video images were played back frame by frame, and the phase at the beginning of each gait cycle was confirmed. One gait cycle is defined as the movement starting from initial contact on one side till the next initial contact on the same side. The beginning of the loading response phase is defined as the initial contact, the beginning of mid stance is defined as opposite side toe off, the beginning of terminal stance is defined as heel off, the beginning of pre swing is defined as opposite side initial contact, the beginning of initial swing is defined as toe off, the beginning of the mid swing is defined as intersection of the feet, and the beginning of the terminal stance is defined as the vertical lower leg.

In the 10 m-walk test, the patient used ankle foot orthosis preoperatively, and did not use orthosis pre- and post-HAL intervention. He used the walker as a walking aid.

Table 1 shows the results of the 10 m-walk test. Speed, stride, and cadence for walking were almost the same preoperatively and pre-HAL intervention. Post-first HAL intervention, speed, stride, and cadence were increased compared to pre-HAL intervention. This was maintained until the next HAL intervention and further improved after the second HAL intervention. HAL intervention increased walking speed from 21.7 to 32.1 m/min, stride from 0.40 to 0.47 m, and cadence from 54.23 to 68.9 steps/min.

**Table 1:** 10-meter walk test

	Speed (m/min)	Stride (m/step)	Cadence (steps/min)
Pre-operation	21.26	0.38	55.28
Pre-first HAL intervention	21.71	0.4	54.28
Post-first HAL intervention	24	0.41	58.8
Pre-second HAL intervention	26.71	0.45	59.81
Post-second HAL intervention	36.05	0.47	68.86

*HAL* Hybrid Assistive Limb

**Fig. 2:**
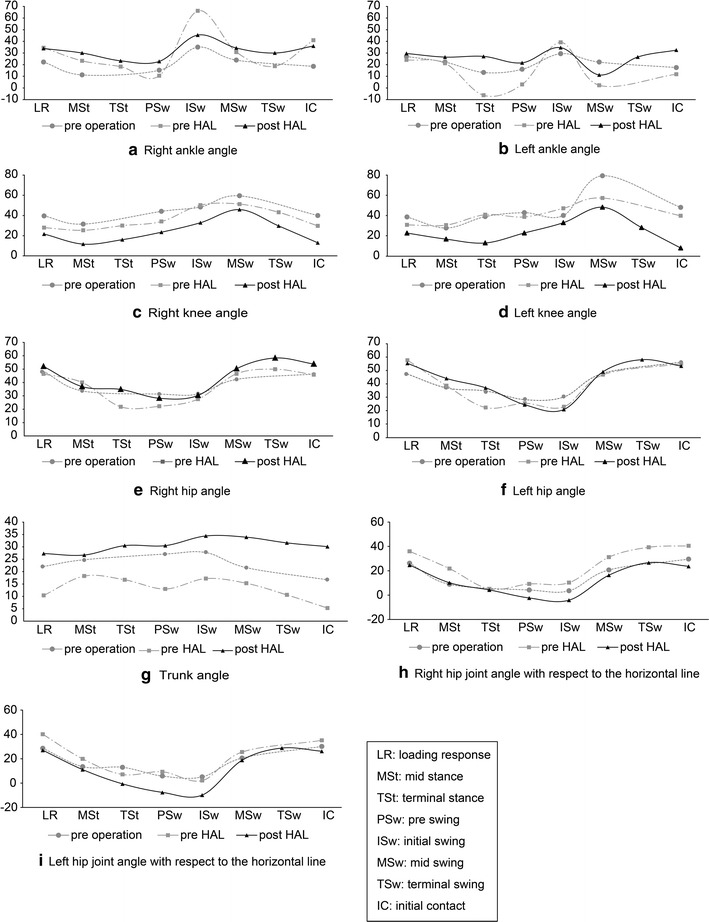
Joint angles pre-surgery, pre-first HAL, and post-second HAL. **a** Right and **b** left ankle, **c** right and **d** left knee, **e** right and **f** left hip, **g** trunk, and **h** right and **i** Left hip joint angle with respect to the horizontal line. *LR* loading response, *Mst* mid stance, *Tst* terminal stance, *PSw* pre-swing, *Isw* initial swing, *MSw* mid swing, *TSw* terminal swing, *IC* initial contact

**Fig. 3:**
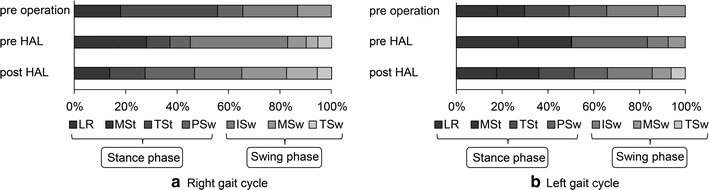
Gait cycle. **a** Right side, **b** left side. *LR* loading response, *Mst* mid stance, *Tst* terminal stance, *PSw* pre-swing, *Isw* initial swing, *MSw* mid swing, *TSw* terminal swing

Ankle angle during walking showed little change because he wore a short leg brace while walking preoperatively. Pre-HAL intervention, the dorsiflexion angle was large in the terminal stance and pre-swing, the plantar flexion angle was large in the initial swing, and there was a large difference between the right and left ankle angle (Fig. 2a, b). Post-HAL intervention, range of movement of the ankle joint became more narrow, left-right symmetry increased (Fig. 2a, b), and extension angles of the knee in the stance phase increased (Fig. 2c, d). Excessive dorsiflexion and plantar flexion of the ankle joint disappeared due to extension of the angle of the knee (Fig. 2a, b). His extension angle of the hip did not increase and the trunk was leaning forward (Fig. 2e, f). Flexion of the upper limbs was strong due to spastic diplegia; therefore, he walked with the head bent forward. Because of the anteversion position, his hip joint did not show extension in walking (Fig. 2g). Although the horizontal plane and the hip joint were examined, extension of the hip angle in the pre-swing phase was increased (Fig. 2h, i). In the gait cycle, asymmetry between the left and right was prominent preoperatively (Fig. 3). Comparing pre- and post-HAL, the proportion of terminal stance in the stance phase and swing phase was increased. Since the length of stride was expanded due to increased extension angles of the knee and hip, the ratio of terminal stance and terminal swing increased (Fig. 3).

After HAL intervention, he was able to crawl and gained the ability to move within his house, similar to his status before operation. There was no adverse event such as fall or fractures during the HAL intervention. The patient was able to walk easily while wearing HAL, and he actively participated in HAL intervention.

## Discussions and conclusions

Before the operation, a crouching gait was prominent, with noticeable flexion contracture of the knee. Although the extension angle of the knee expanded postoperatively, the crouching gait remained. The crouching gait improved due to the expanded extension angle of the knee in the stance phase after the HAL intervention. With increased extension angles of the knee and hip in the stance phase, the speed, length of stride for walking, and cadence markedly increased. Based on these findings, we report that the combination of HAL intervention and surgery with improvement of contractures of the knee and ankle can enhance improvement in walking ability for the patient with cerebral palsy. Improvement in walking ability leads to improved lower limb function, improved standing ability, and reduced need for assistance.

After the first HAL intervention, speed, length of stride for walking, and cadence increased on the 10 m-walk test in the pre-second HAL intervention. We considered this a sustained effect of the first intervention. Post-second HAL intervention, the speed, length of stride for walking, and cadence increased more than before the second intervention. The result of this intervention suggested that the effect is potentially persistent and the effect is increased with repeated HAL interventions. Thus, it is necessary to consider the frequency and timing of HAL therapy.

On video gait analysis using Dartfish, it is possible to determine the gait cycle accurately [19] and to measure the joint angle based on the timing of the gait cycle. Three-dimensional gait analysis is necessary for more accurate joint angle measurement.

Cerebral palsy is the most frequent motor disability of childhood, with a neonatal prevalence of approximately 2 per 1000 live births [20]. Robot rehabilitation for cerebral palsy has been described in several papers [21–25]. After a 3-week trial of robotic-assisted treadmill therapy, patients with bilateral spastic cerebral palsy showed improvements in the functional tasks of standing and walking [23]. Retraining gait using the driven gait orthosis improved gait speed and the Gross Motor Function Measure score [24]. Repeated active movement aligns with the motor learning theory currently popular in physical treatment as a means of inducing neuroplastic changes in the brain [26]. Robot intervention for physical treatment is considered to be effective in improving walking ability for patients with cerebral palsy.

Hybrid Assistive Limb intervention may be a new treatment option in patients with cerebral palsy after soft tissue surgery. To obtain a treatment effect, it is necessary to investigate the indications and limitations of HAL intervention, and to determine an effective protocol using HAL intervention. Borggraefe et al. [22, 23] aimed for 12 sessions of robot intervention (4 sessions/week). Meyer et al. [24] aimed for 20 sessions of robot intervention (2–5 sessions/week). The currently reported case includes less frequent training than previous reports. In children and young people, the effect of this intervention may be obtained with less frequent application.

These robots provide autonomous motion to patients based on the desired kinematic trajectory of the lower limb joints or the end effector, mimicking the walking motion of an able-bodied person. HAL interactively provides motion according to the wearer’s voluntary drive [1].

In this case, although the flexion contractures of the bilateral knees and equinus foot improved, the muscle strength with bilateral knee flexion and ankle dorsiflexion was decreased following tendon lengthening of the bilateral hamstrings and Achilles tendons. HAL intervention can be an effective tool for improvement in range of movement and muscle strength after soft tissue operations by providing assistance with the power units of the HAL hip and knee joints. HAL intervention may be applied in patients with cerebral palsy who undergo soft tissue operations for improvement in joint contractures and decreased muscle strength.

After myotomy, tenotomy, or the lengthening of tendon or muscle, muscles cannot perform sufficiently, and the alignment of the legs is altered. HAL can assist the muscle and provide walking training to improve towards a more normal gait.

Voluntary drive and normalized motion assistance provided by the external device form the foundation for a proprioceptive feedback loop for patients with cerebral palsy. HAL intervention produces neural activity and repeated execution of specific tasks. The HAL intervention promotes learning and leads to a state of appropriate proprioceptive feedback. We were able to carry out the HAL intervention for cerebral palsy in a postoperative patient safely and effectively.

After soft tissue surgery for cerebral palsy, although flexion contractures of the bilateral knees and equinus foot improved, muscle strength of bilateral knee flexion and dorsiflexion temporarily decreased in our patient. In this case, using HAL intervention, the extension angle of the knee and hip in the stance phase was expanded and the walking speed, length of stride, and cadence increased. Use of the HAL intervention in a postoperative patient with cerebral palsy can enhance improvement in walking ability.

We will increase the use of HAL for patients with spastic cerebral palsy. Patients with cerebral palsy may have thin legs, in which case it is necessary to adjust the size of the cuff and belt for the lower limbs, because the size of the cuff and belt is large. Meyer uses Lokomat for patients with Guillain–Barre syndrome and athetoid-type cerebral palsy. We will consider using HAL for patients with other types of cerebral palsy. Further studies are needed to examine the safety and potential applications of this technique.

## Additional files

**Additional file 1.** Image of Hybrid Assistive Limb (HAL^®^) intervention for cerebral palsy.

**Additional file 2.** Video of Hybrid Assistive Limb (HAL^®^) intervention for cerebral palsy.

**Additional file 3.** Image of Gait pre and post Hybrid Assistive Limb (HAL^®^) intervention for cerebral palsy.

**Additional file 4.** Video of gait pre and post Hybrid Assistive Limb (HAL^®^) intervention for cerebral palsy.

## Abbreviations

CAC: cybernic autonomous control
CVC: cybernic voluntary control
DKE: dorsiflexion with knee extension
DKF: dorsiflexion with knee flexion
HAL: Hybrid Assistive Limb
SCI: spinal cord injury
