# Supplementary figures and images for: Use of Hybrid Assistive Limb (HAL®) for a postoperative patient with cerebral palsy: a case report
Source: BMC Res Notes. 2018 Mar 27;11:201. doi: 10.1186/s13104-018-3311-z (PMC5870205; doi:10.1186/s13104-018-3311-z)

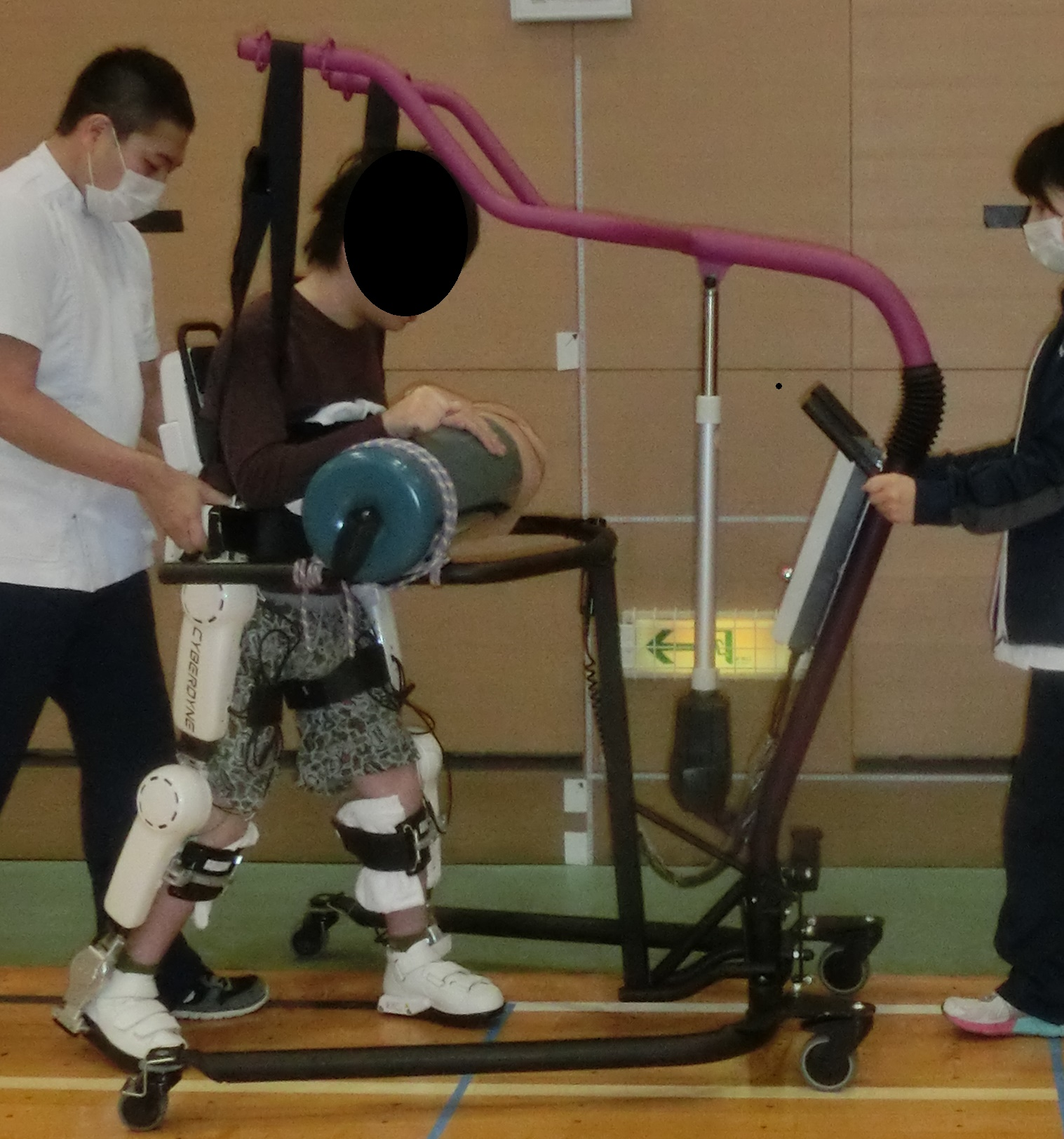

Supplement: Supplementary file 1 — Additional file 1. Image of Hybrid Assistive Limb (HAL®) intervention for cerebral palsy. [file 13104_2018_3311_MOESM1_ESM.tif]

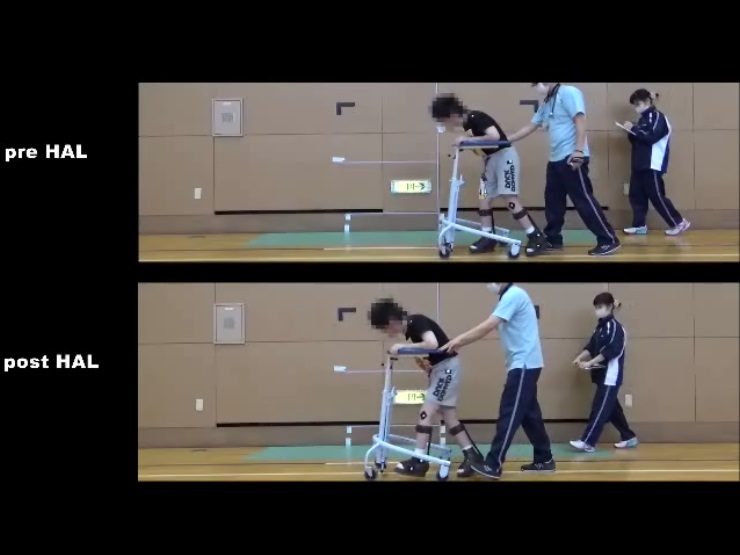

Supplement: Supplementary file 3 — Additional file 3. Image of Gait pre and post Hybrid Assistive Limb (HAL®) intervention for cerebral palsy. [file 13104_2018_3311_MOESM3_ESM.jpg]
